# Poly(amidoamine)-Cholesterol Conjugate Nanoparticles Obtained by Electrospraying as Novel Tamoxifen Delivery System

**DOI:** 10.1155/2011/587604

**Published:** 2011-06-07

**Authors:** R. Cavalli, A. Bisazza, R. Bussano, M. Trotta, A. Civra, D. Lembo, E. Ranucci, P. Ferruti

**Affiliations:** ^1^Dipartimento di Scienza e Tecnologia del Farmaco, Università di Torino, Via P. Giuria 9, 10125 Torino, Italy; ^2^Department of Clinical and Biological Sciences, University of Torino, Orbassano, 10043 Torino, Italy; ^3^Dipartimento di Chimica Organica e Industriale, Università degli Studi di Milano, Via Venezian 21, 20133 Milano, Italy

## Abstract

A new poly(amidoamine)-cholesterol (PAA-cholesterol) conjugate was synthesized, characterized and used to produce nanoparticles by the electrospraying technique. The electrospraying is a method of liquid atomization that consists in the dispersion of a solution into small charged droplets by an electric field. Tuning the electrospraying process parameters spherical PAA-chol nanoparticles formed. The PAA-cholesterol nanoparticles showed sizes lower than 500 nm and spherical shape. The drug incorporation capacity was investigated using tamoxifen, a lipophilic anticancer drug, as model drug. The incorporation of the tamoxifen did not affect the shape and sizes of nanoparticles showing a drug loading of 40%. Tamoxifen-loaded nanoparticles exhibited a higher dose-dependent cytotoxicity than free tamoxifen, while blank nanoparticles did not show any cytotoxic effect at the same concentrations. The electrospray technique might be proposed to produce tamoxifen-loaded PAA-chol nanoparticle in powder form without any excipient in a single step.

## 1. Introduction

Polymeric nanoparticles focused a great attention in the biomedical field as delivery systems for active molecules. These nanoparticles have the potential to act as a reservoir of drugs, protecting them from the environments and controlling their release rates, thereby enhancing the biological activity and decreasing the adverse side effects [1–4].

Various procedures have been proposed in the literature for the fabrication of polymeric nanoparticles and the most used are based on emulsion techniques. However novel methods are attracting increasing attention. One of such is electrohydrodynamic atomization (EHDA), a process with many applications, such as manufacturing nanoscale polymer fibres or thin film and particulate systems [5, 6]. Particularly, EHDA in the cone jet mode (electrospraying) has been previously studied to produce polymeric particles which can be used as drug delivery systems [7, 8]. The electrospray is a method of liquid atomization that consists in the dispersion of a solution into small charged droplets by an electric field. 

Electrospraying is a one-step technique with the potential to ensure particle with reproducible sizes and morphology with a narrow size distribution in the micro- and nanometer range by selecting the proper process conditions.

The principle of electrospraying is based on the capacity of an electric field to deform the interface of a droplet as reported by Jaworek [9]. Particularly, when an electric field is applied on a droplet, it generates an electrostatic force inside the droplets able to overcome the cohesive force of the droplet. Thus the droplet will undergo break-up into smaller droplets in the micro-nanoscale range. Depending on the spraying mode, droplets can be smaller than 100 nm with low standard deviation. This charged aerosol is self-dispersing preventing the droplets from coalescence. This phenomenon is known as Taylor Cone and consists in the progressive shrinking of the charged droplet into a cone from which smaller charged droplets will be ejected [10]. 

The electrospraying process is simple; it consists in the loading of a polymer solution in a syringe which is infused at constant rate by a pump through a highly charged capillary, forming a droplet at the tip. A dropled formed at the capillary tip and after the droplet detached from the Taylor cone the solvent evaporates generating solid particles. During the electrospraying process there are several parameters which can affect particle sizes, size distribution, encapsulation efficiency, and* in vitro *release profiles. These include voltage, flow rate, distance from the collector, solvent, and needle gauge. Consequently the number of parameters to be considered to obtain a reproducible process is several and the optimization is complex. 

Previously we have tuned the electrospraying process parameters for producing lipid-based micro-nanoparticles [11]. Narrowly dispersed spherical particles with sizes lower than 1 *μ*m were obtained using stearic acid and ethylcellulose in a 4.5 : 0.5 ratio (w/w). 

The aim of this work was to investigate the feasibility of obtaining solid polymeric nanoparticles using a cholesterol polyamidoamines (PAAs) conjugate by electrospraying. 

PAAs are synthetic degradable polymers obtained by Micheal-type polyaddition of primary or bis-secondary amines to bis-acrylamides [12]. All PAAs contain amide and tertiary amine groups along the main chain.

In PAA-cholesterol conjugates an active substituent was bound to the polymer chain through a disulfide linkage that is known to be stable in the bloodstream but amenable to reductive cleavage inside cells. Preliminary cytocompatibility tests demonstrated that all prepared PAA-cholesterol samples are cytocompatible and thus show potential for biotechnological and pharmaceutical applications [13]. In this work a new PAA conjugate was used to prepare solid nanoparticles by electrospraying as potential drug delivery systems. The goal of the work was to develop a reproducible one-step process to obtain spherical solid PAA-cholesterol nanoparticles with homogeneous size distribution by electrospraying. Moreover cytotoxicity of nanoparticles was assessed in order to avoid the possibility of toxic residues after the electrospraying process.

Tamoxifen, a lipophilic anticancer drug, was used as model drug to study the encapsulation capacity of the PAA conjugate. Tamoxifen is a selective estrogen receptor modulator widely used in breast cancer therapy. The drug can produce serious side effects, as thrombosis, pulmonary embolism, and modification in liver enzyme levels. In addition cancer cells can develop resistance against tamoxifen, and it may initiate endometrial cancer. The encapsulation of tamoxifen in a drug delivery system might provide a better drug release profile potentially preventing the development of cell resistance [14].

## 2. Material and Methods

### 2.1. Material

n-Pentanol was from Merck (Darmstadt, Germany). Tamoxifen, sodium citrate, Rhodamine B, and citric acid were from Sigma Aldrich (St. Louis, USA). Cellulose dialysis membrane (Spectra/Por dialysis membrane) was from Spectrum Laboratories, Inc (Canada). 2,2-bis(acrylamido)acetic acid and 1,4-bis(acryloil)piperazine were synthesized as previously described [15, 16]. All reagents are of analytical grade.

### 2.2. Cells

Both MCF-7 (a human breast adenocarcinoma cell line) and Vero (an African green monkey kidney cell line) were maintained in Minimum Essential Medium (PAA, 4061 Pasching, Austria) with 10% Fetal Calf Serum (Gibco/BRL) and 1% Zell Shield (Minerva Biolabs GmbH, Berlin). Subculturing of cells was carried out by trypsinization and by diluting cells with fresh medium. Cells were grown in the presence of 5% CO_2_ at 37°C.

### 2.3. Electrospraying Setup

**Figure 1:**
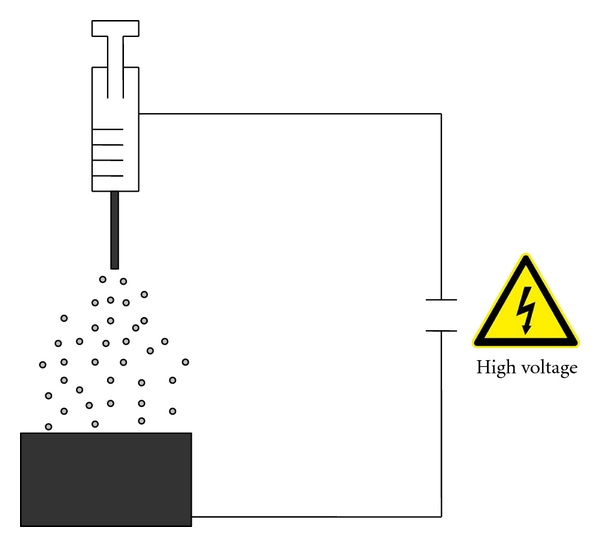
Scheme of the apparatus for electrospraying.

The apparatus for electrospraying comprises a 2.5 mL syringe connected to an infusion pump (KDS 100, Biological Instruments, VA, Italy). A Teflon pipe connects the syringe to the tip of a metal capillary (ID: 0.6 mm) (Figure 1). An aluminium foil collector is placed opposite the capillary as counter electrode. A strong electric field was applied between the tip and the collector. The distance from metal tip to collecting plate varied from 20 to 15 cm.

The solution contained in the syringe is supplied to the nozzle at a flow rate forming a droplet. The electric field induces charges on the droplet surface. A liquid jet occurs that can break up in droplets with a narrow size distribution. Solid particles formed by solvent evaporation from the droplets which travel through the electric field. 

### 2.4. Preparation and Characterization of PAA-Cholesterol Conjugate

**Figure 2:**
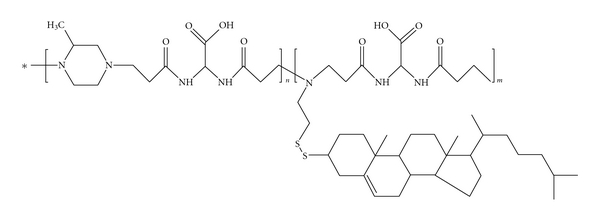
Chemical structure of the PAA-cholesterol conjugate.

A PAA-cholesterol conjugate derived from two different bis-acrylamides, namely 2,2-bis(acrylamido)acetic acid and 1,4-bis(acryloil)piperazine, with a cholesterol content of 8.1% w/w and *M*_*w*_ = 13000 was obtained (Figure 2). The reaction pathway consisted of three steps: (1) the synthesis of a PAA-based hydrogel containing cystamine as cross-linker, (2) a disulfide-exchange reaction with 2,2′-dipyridyl disulfide that leads to soluble linear polymers containing ethenyldithiopyridine moieties, and (3) a thiol-exchange reaction between thiocholesterol and the dithiopyridine moieties [13].

The solubility of the PAA-cholesterol conjugate was determined in water and in n-pentanol the solvent selected for the electrospraying. The surface tension of the polymer solutions in water was measured using a Kruss Ring platinum tensiometer K10 (Hamburg).

The zeta potential (ZP) values of the conjugate were determined in aqueous solutions at increasing pH values, ranging from 4.0 to 7.0, to verify the polymer charge distribution as function of the pH. A 90 Plus instrument (Brookhaven, NY, USA) was used to determine the electrophoretic mobility and the zeta potential of the polymer. For the determinations, the aqueous solutions of the polymer were placed in the electrophoretic cell, where an electric field of about 14 V/cm was applied. Each value reported is the average of ten measurements. The electrophoretic mobility measured was converted into Zeta Potential using the Smoluchowski equation [17].

The PAA-solution conductibility in water was determined using a conductometer (Orion, Boston, USA).

### 2.5. Hemolytic Activity Determination of PAA-Cholesterol Conjugate

The haemolytic activity of the PAA-cholesterol conjugate was evaluated on human blood. Different percentages (2%, 4%, 7%, 10%, and 15% w/v) of polymer were added in a erythrocytes suspension (30% v/v) phosphate buffer, pH 7.4. A sample containing only a suspension of erythrocytes (30% v/v) in phosphate buffer pH 7.4 was used as blank. In addition a blank sample containing an excess of ammonium chloride was prepared to obtain complete erythrocyte hemolysis as hemolytic control.

After 90 minutes of incubation at 37°C the samples were centrifuged at 1500 rpm for 10 minutes and the supernatant was analyzed using a Lambda 2 Perkin-Elmer spectrophotometer at a wavelength of 543 nm. The percentage of hemolysis was calculated versus the 100% hemolysis control.

### 2.6. Preparation of PAA-Cholesterol Nanoparticles

To prepare PAA-cholesterol nanoparticles the electrospraying apparatus previously described was used. Preliminary experiments were carried out to select the process parameters suitable to obtain spherical nanoparticles with the PAA-cholesterol conjugate. Different parameters were varied to tune the process; flow rate of 15, 10, and 5 *μ*L min^−1^ and electric field of 20, 25, and 30 KV were mainly investigated. The experimental conditions selected were a flow rate of 5 *μ*L min^−1^ and an electric field of 20 KV applied between the capillary tip and an aluminum plate. The selected distance from metal tip to collecting plate was 15 cm. A solution of PAA-cholesterol conjugate in n-pentanol (1% w/w) was prepared and supplied to the capillary nozzle with a 5 *μ*L min^−1^ flow. During free flight the organic solvent evaporated and solid nanoparticles collected on the plate. To obtain fluorescent-labelled nanoparticles Rhodamine B was added in the polymer pentanol solution (0.05% w/v). The same process parameters were applied during electrospraying.

### 2.7. Preparation of Tamoxifen-Loaded PAA-Cholesterol Nanoparticles

Tamoxifen-loaded PAA-cholesterol nanoparticles were prepared by dissolving the drug (5 mg/mL) in the conjugate n-pentanol solution (10 mg/mL) under stirring. The solution was then electrosprayed to obtain the drug loaded PAA-cholesterol nanoparticles using the same process parameters selected to obtain blank nanoparticles. 

### 2.8. Quantitative Determination of Tamoxifen

The amount of tamoxifen-loaded into the nanoparticles was determined after addition of 2.0 mL of phosphate buffer pH 7.4 containing 20 mg of citric acid to a weighed amount of drug-loaded nanoparticles (2 mg). After vortex and centrifuge for 5 minutes at 5000 rpm, 2 mL of ethanol and 0.5 mL of water were added to the precipitate. After stirring and separation the supernatant was analyzed by HPLC.

Tamoxifen content was determined using an HPLC system consisting of Shimadzu liquid chromatograph (Shimadzu, Kyoto, Japan) equipped with an SDP 10A variable wavelength ultraviolet detector and a CR6A integrator. A Lichrospher C-18, 5 *μ*m (Merck, Darmstadt, Germany), 25 cm × 4.6 mm ID reversed-phase column was used. The column was eluted with a mobile phase containing methanol/water/triethylamine (89/11/1, v/v). The eluent was run at rate of 1 mL/min and monitored at 265 nm following injected volumes of 20 *μ*L of tamoxifen standard solutions and samples. The calibration curve was found to be linear in the range 0.05–30 *μ*g/mL. Each sample was analyzed in triplicate.

### 2.9. Characterization of the PAA-Cholesterol Nanoparticles

The average diameters and polydispersity indices of the three nanoparticle formulations were determined after dispersion of the samples in filtered water by photocorrelation spectroscopy (PCS) using a 90 Plus instrument (Brookhaven, NY, USA) at a fixed angle of 90° and a temperature of 25°C. The polydispersity index indicates the size distribution within a nanoparticles population. The electrophoretic mobility and zeta potential of the formulations were determined using a 90 Plus instrument (Brookhaven, NY, USA). For zeta potential determination, samples of the formulation were placed in the electrophoretic cell, where an electric field of about 15 V/cm was applied. Each sample was analyzed at least in triplicate. The electrophoretic mobility measured was converted into zeta potential using the Smoluchowski equation [17].

The nanoparticles morphology was evaluated by Scanning Electron Microscopy (SEM) (Leica Stereoscan 410, Wetzlar, Germany) and fluorescent microscopy. To perform SEM a thin layer of particles was mounted on a copper stud, which was then sputter coated with gold (SCD 050, Lewica, Wetzlar, Germany) for 60 seconds under vacuum at a current intensity of 60 mA. The gold-coated particle layer was scanned using the accelerating voltage scanning of 20 kV.

### 2.10. Thermal Analysis of Nanoparticles

Differential scanning calorimetry (DSC) analysis was carried out using a DSC7 differential scanning calorimeter (Perkin-Elmer, Conn, USA) equipped with a TAC7/DX instrument controller. The instrument was calibrated with indium for melting point and heat of fusion. A heating rate of 10°C/min was employed in the 30–120°C temperature range. Standard aluminium sample pans (Perkin-Elmer) were used; an empty pan was used as reference standard. Blank nanoparticles, tamoxifen-loaded nanoparticles, and tamoxifen powder (3-4 mg) were weighed in conventional aluminium pans, and analyses were performed under nitrogen purge; triple runs were carried out for each sample. 

### 2.11. In Vitro Release Kinetics of Tamoxifen

A multicompartmental rotating cell was used to evaluate the *in vitro* release profile of tamoxifen. A tamoxifen aqueous suspension 1.17 mM as control and tamoxifen-loaded nanoparticles at the same concentration dispersed in water were prepared, and 1 mL of each was placed in the donor compartment. A cellulose dialysis membrane with cutoff at 12,000–14,000 was chosen to separate the compartments. The receptor compartment was filled with 1 mL of pH 5.5 0.1 M citrate buffer. Each experiment lasted 24 h. At fixed times, the receptor buffer was completely withdrawn and replaced with fresh citrate buffer. The withdrawn samples were then analyzed by HPLC. The experiment was performed in triplicate.

### 2.12. Internalization Study of PAA-Cholesterol Nanoparticles

The cellular uptake of PAA-cholesterol fluorescent nanoparticles was evaluated through confocal laser scanning microscopy on Vero cell. Exponentially growing cells were plated and cultured overnight in 24-well plates on glass coverslips; the cell monolayers were incubated with appropriated dilutions of PAA-cholesterol fluorescent nanoparticle suspension for 1 h and then extensively washed with PBS for observation of live cells. Confocal sections were taken on an inverted Zeiss LSM510 fluorescence microscope.

### 2.13. Cytotoxicity Assay

To test the cytotoxic effect of tamoxifen-loaded nanoparticles, MCF-7 cells were seeded in a 96-well plate at a density of 1.2 × 10^4^/well; the next day they were treated with increasing concentrations, ranging from 1 to 40 *μ*M, of free tamoxifen and tamoxifen-loaded nanoparticles. Treatment with equal concentrations of blank nanoparticles was made in order to rule out the possibility of any cytotoxic effect ascribable to the delivery system.

After 24, 48, and 72 hours of incubation, cell viability was determined by the CellTiter 96 Proliferation Assay Kit (Promega, Madison, Wls, USA) according to the manufacturer's instructions. Absorbances were measured using a Microplate Reader (Model 680, BIORAD) at 490 nm. The effect on cell viability of the formulation at different concentrations was expressed as a percentage, by comparing treated cells with cells incubated with culture medium alone. The 50% cytotoxic concentration (CC_50_) values and the 95% confidence intervals (CIs) were determined using the Prism software (GraphPad Software, San Diego, CA).

## 3. Results

Firstly the new PAA-cholesterol conjugate was *in vitro* characterized. The percentage of cholesterol was determined by NMR resulting in 8% w/w.

For the electrospraying process is necessary an organic solvent in which the PAA-Cholesterol conjugate is very soluble. For this purpose 1-pentanol (b.p. = 137.5°C) in which the conjugate is soluble more than 2% w/v was selected. 

**Figure 3:**
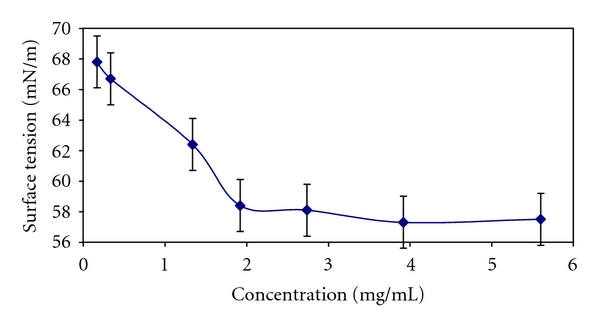
Surface tension of PAA-cholesterol conjugate in water.

The conjugate is amphiphilic for the presence of cholesterol in the structure. The surface tension of the PAA-cholesterol conjugate in water at pH 6.0 was determined, and it is reported in Figure 3. PAA-cholesterol showed a CMC in water of about 2 mg/mL.

Zeta potential measurements demonstrated that PAA-cholesterol is positively charged in aqueous solution with a value of +21 mV at pH 7.0 and the positive charge increase lowering the pH value to 4.0. The conjugate conductibility in water was 74.8 *μ*S.

No significant haemolytic activity was observed for PAA-cholesterol conjugate after 90 minutes of incubation in blood at pH 7.4 up to a concentration of 15% w/v.

Spherical solid PAA-cholesterol nanoparticles formed easily using an electric field of 20 KV and a flow of 5 *μ*L min^−1^. The nanoparticle sizes were mainly tuned by the control of electrospraying flow rate and polymer concentration. 

The physicochemical characteristics of PAA-cholesterol nanoparticles are reported in Table 1.

All the PAA-cholesterol conjugate nanoparticles showed sizes lower than 500 nm with a quite narrow size distribution and a positive Zeta Potential. The loaded nanoparticles had sensible greater sizes and a decrease of the Zeta potential values demonstrating the presence of incorporated molecules in the nanoparticle structure.

**Figure 4:**
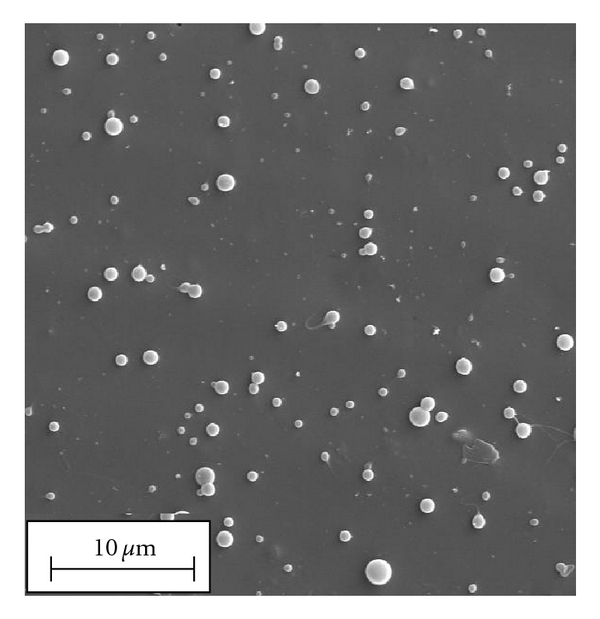
SEM image of blank PAA-cholesterol nanoparticles.

SEM analyses showed that PAA-cholesterol nanoparticles are spherical with smooth surfaces and confirmed their sizes. The blank PAA-cholesterol nanoparticles image is reported in Figure 4. 

**Figure 5:**
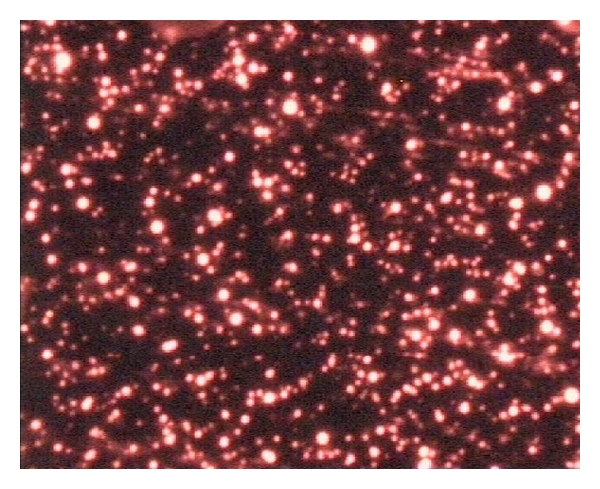
Fluorescent PAA-cholesterol nanoparticles containing Rhodamine B (fluorescent microscopy).

**Figure 6:**
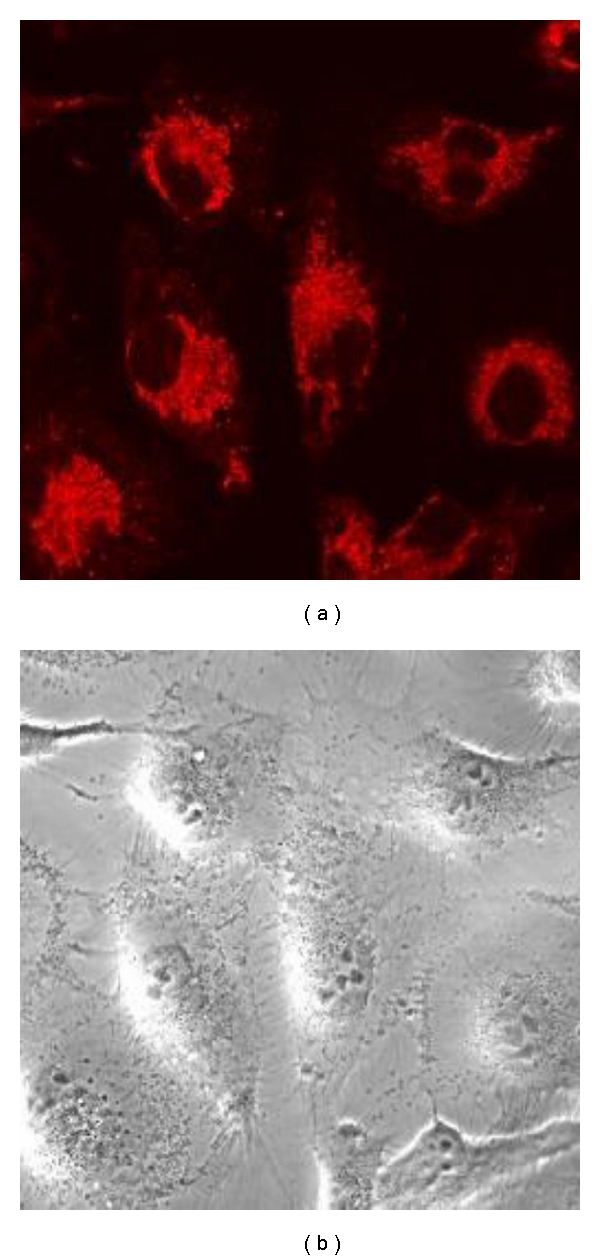
Internalization of fluorescent PAA-cholesterol nanoparticles on Vero cells analysed by confocal laser scanning microscopy.

A fluorescent PAA-cholesterol formulation was also prepared by the electrospraying process using Rhodamine B as fluorescent marker to evaluate the nanoparticle cell internalization. The morphology of the fluorescent formulation is reported in Figure 5. The fluorescent nanoparticles were easily internalized in Vero cells (Figure 6).

SEM analysis confirmed sizes and shape of tamoxifen-loaded PAA-cholesterol nanoparticles.

**Figure 7:**
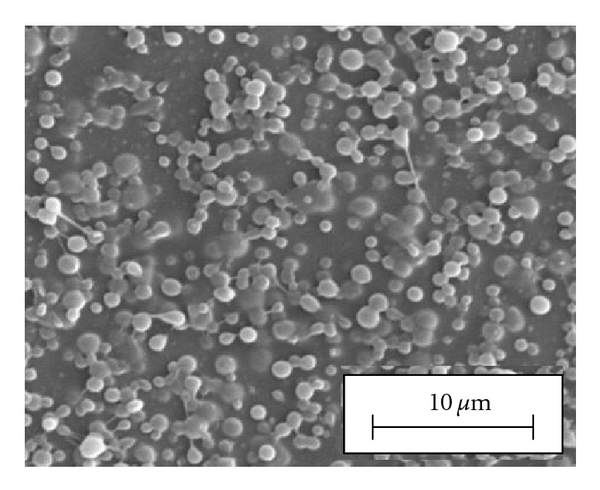
SEM image of *In vitro *release of the formulations.

The incorporation of the drug did not affect the shape and the smooth surface of nanoparticles as shown in Figure 7.

As it is possible to note in the figure the presence of the drug could affect the physicochemical characteristics of the PAA-cholesterol solution favouring a partial coalescence of the droplet. Decreasing the amount of tamoxifen, well-separated nanoparticles were obtained (data not shown).

Tamoxifen-loaded PAA-cholesterol nanoparticles showed a drug loading of about 40% w/w and the encapsulation efficiency of about 90%.

**Figure 8:**
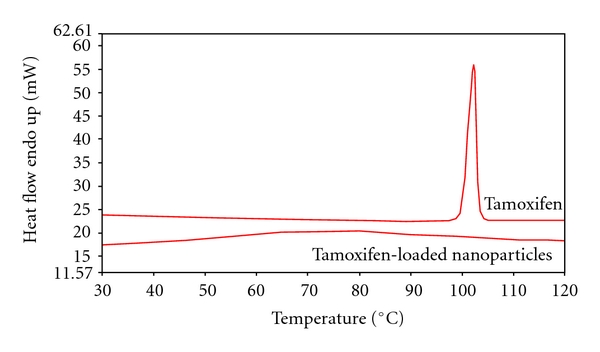
Thermal analysis of tamoxifen and tamoxifen-loaded nanoparticles.

Thermal analysis of tamoxifen-loaded nanoparticles did not show the endothermic peak at about 97°C related to the drug melting. The disappearance of the melting peak in the DSC profile indicates that the drug can be dispersed in the polymer matrix. The PAA-cholesterol conjugate did not show thermal change in this temperature range. The DSC thermograms of the tamoxifen-loaded nanoparticles and of tamoxifen are reported in Figure 8.

**Figure 9:**
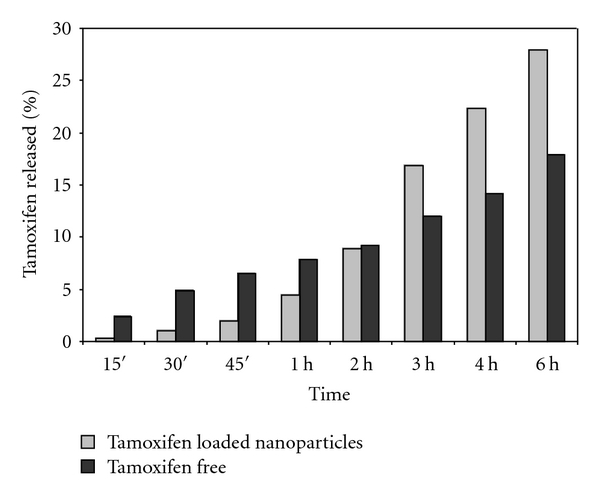
*In vitro* release of tamoxifen from the formulations.

The *in vitro* release profile of tamoxifen from the drug-loaded nanoparticle showed a slow release over time without initial burst effect indicating that the drug is mainly incorporated in the PAA-cholesterol matrix and not adsorbed on particle surface. After 6 h about 26% of tamoxifen was released from the PAA-cholesterol nanoparticles. On the contrary about 15% of tamoxifen from aqueous drug suspension diffused after 6 h (Figure 9).

### 3.1. Cytotoxicity Assay

To assess the activity of the formulation, MCF-7 cells were incubated with solutions containing blank nanoparticles or tamoxifen-loaded nanoparticles, having the same nanoparticle concentration. Free tamoxifen was used as positive control. As free tamoxifen was diluted in DMSO, corresponding volumes of DMSO were also added for comparison. After 24, 48, and 72 hours from the beginning of treatment, cells were analyzed by MTS colorimetric assay to test cell viability. 

**Figure 10:**
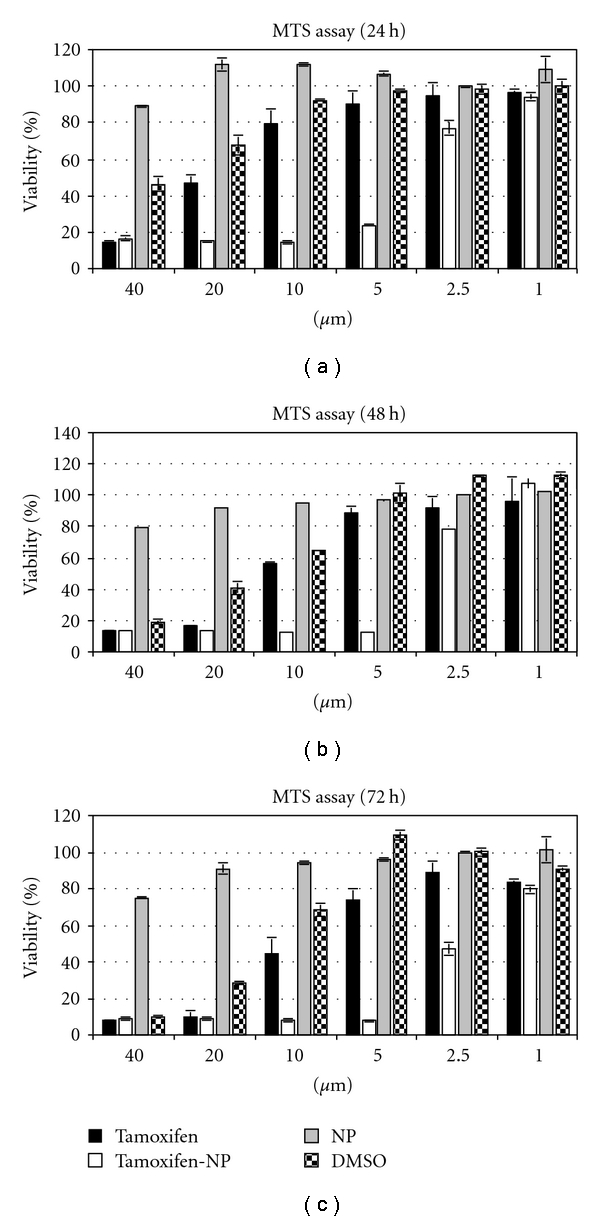
Cell viability of MCF-7 breast cancer cells incubated for 24, 48, and 72 h (a, b, and c panels, resp.) with free tamoxifen (Tamoxifen), tamoxifen-loaded nanoparticles (tamoxifen-NP), empty nanoparticles (NP), or DMSO. Each bar represents the mean of three samples ± SD.

As shown in Figures 10(a), 10(b), and 10(c), tamoxifen-loaded nanoparticles exhibited a more pronounced concentration-dependent cytotoxicity than free tamoxifen at each time point analyzed. The finding that blank nanoparticles did not show any cytotoxic effect even at high concentrations rules out their contribution to the increased activity of the formulation and confirmed the absence of residues. 

Notably, cytotoxic effect measured for those samples that received volumes of tamoxifen greater than 2 *μ*L (2% v/v) seems mostly ascribable to the presence of DMSO, as indicated by the treatment with this solvent alone.

## 4. Discussion

Electrospraying (electrohydrodynamic spraying) is a process of simultaneous droplet generation and charging by means of electric field [9]. Production of particles of uniform size can be accomplished by generation of cone-jet mode. This mode of spraying is very sensitive to any change in liquid properties, and the droplet size can vary unexpectedly with parameter changes. By the selection of suitable process parameters the production in a single step of solid nanoparticles using a PAA-cholesterol conjugate as matrix was possible. The new PAA conjugate containing 8% of cholesterol is soluble either in water or in organic solvents with a CMC of about 2 mg/mL. Because of its amphiphilic nature it is particularly suitable to produce nanoparticles by electrospraying [13]. The surface tension of the solution can affect the nanoparticle formation; generally it was shown that, decreasing the surface tension of a solution, there is a decrease in the particle sizes with a corresponding increase in standard deviation of the particle sizes distribution [18]. Surface charge density and surface tension play important roles in the process. When the surface charge density is low, the Rayleigh limit, the maximum limit of surface charge density when the electrostatic forces exceed surface tension, is never reached. Another possibility is that the surface charge density of the droplets is high, so the Rayleigh limit is reached immediately or after solvent evaporation and droplets disintegrate (Coulomb fission), forming small charged droplets. In the process the Coulomb fission should be avoided because droplets of uniform size are required [11, 19]. The PAA-cholesterol nanoparticles are positive charged showing that during the droplet shrinking the hydrophilic region remains on the surface, while the cholesterol molecules can be oriented inside the droplets.

Reproducible PAA-cholesterol nanoparticles with sizes lower than 300 nm and spherical shape from pentanol solution were obtained in one single step.

The effect of conductivity on particle formation has also been investigated [20]. The increase of a solution conductivity from *μ*S/cm to mS/cm resulted in a marked reduction of the particle size due to the Coulomb fission.

Tamoxifen is a hydrophobic molecule with a low water solubility (0.4 mg/mL), a high hygroscopicity and UV light sensitivity. Its solubility in pentanol reached 2 mg/mL. Tamoxifen-loaded PAA-cholesterol nanoparticles formed by electrospray maintaining a mean diameter lower than 300 nm. The drug is incorporated in the polymer matrix as DSC and *in vitro* release studies showed. The absence of an initial burst effect confirmed the incorporation of tamoxifen inside the polymer matrix. Hydrophobic interaction between cholesterol and hydrophobic portion of tamoxifen molecules could favour the incorporation of the drug in the internal lipophilic region. The drug probably remains molecularly dispersed in the PAA-cholesterol conjugate matrix without crystallizing. The disappearance of the melting peak of the drug in the DSC profile can confirm this hypothesis.

The electrospraying process tuned employs n-pentanol, an organic solvent with boiling point of 137.5°C in which the conjugate is soluble. It is important to assess that this solvent is completely removed otherwise the electrosprayed nanoparticles might be toxic to cells.

Therefore we tested the blank nanoparticles and the results showed no cytotoxicity up to a concentration of 20 *μ*M. This behaviour indicates that the conjugate nanoparticles are not toxic as previously reported [13] and that no n-pentanol remains incorporated in the polymer matrix but it evaporates during the flight and nanoparticle formation. The data are in agreement with pentanol residues determined previously by gas chromatography in the stearic acid:ethylcellulose particles (4.5 : 0.5 w/w). The pentanol content was 0.2 milligrams per gram of lipid based particles [11].

According to previous report [21], we found that MCF7 cells are highly sensitive towards DMSO. Indeed, volumes equal to or higher than 2 *μ*L (2% v/v) result in a cytotoxic effect that partially overlaps the one observed in cells treated with free tamoxifen diluted in DMSO. Therefore, this “background” cytotoxicity leads to an overestimation of the free tamoxifen activity, although the CC_50_ value we measured is comparable to the ones presented in the literature for free tamoxifen diluted in DMSO [22]. By contrast, tamoxifen-loaded PAA-cholesterol nanoparticles showed a clear dose-dependent cytotoxic activity, completely ascribable to the drug. Considering that the CC_50_ value of free tamoxifen is overestimated, the cytotoxic potency of the tamoxifen nanoparticle formulation is even more attractive.

## 5. Conclusion

Solid polymeric nanoparticles with spherical shape and smooth surface were obtained using a new PAA-cholesterol conjugate by electrospraying, a cost effective technique.

In this work electrospraying parameters were tuned to obtain PAA-cholesterol nanoparticles avoiding fiber formation, particularly varying flow rate and voltage applied to the nozzle.

The new PAA-cholesterol conjugate is suitable to produce nanoparticles by electrospraying in the absence of excipients and in powder form in a single step. The PAA-cholesterol nanoparticles show small size, spherical shape, and good drug loading. Tamoxifen-loaded in PAA-cholesterol nanoparticles showed a slow release over time due to the incorporation in the conjugate matrix.

The PAA-cholesterol nanoparticles did not show any toxic effects. The tamoxifen-loaded nanoparticles showed an enhanced cytotoxicity in comparison to the free drug. 

## Figures and Tables

**Table 1:** Characteristics of PAA-cholesterol nanoparticles.

Formulation	d ± SD (nm)	Poly-index	PZ ± SD (mV)
PAA-cholesterol nanoparticles	223.2 ± 10.0	0.29	21.28 ± 2.76

PAA-cholesterol fluorescent nanoparticles	362.8 ± 23.8	0.15	17.89 ± 1.32

PAA-cholesterol tamoxifen nanoparticles	247.0 ± 17.7	0.26	14.86 ± 0.99
